# Endoscopic evaluation of the oral cavity and pharynx: how to do it

**DOI:** 10.1055/a-2436-2082

**Published:** 2024-10-25

**Authors:** Renata Nobre, Noriya Uedo, Ryu Ishihara, Yuki Okubo, James Weiquan Li, Fauze Maluf-Filho

**Affiliations:** 1 Department of Gastroenterology, São Paulo Cancer Institute, São Paulo, Brazil; 2 53312 Department of Gastrointestinal Oncology, Osaka International Cancer Institute, Osaka, Japan; 3 Department of Gastroenterology and Hepatology, Changi General Hospital, Singapore, Singapore

According to the field cancerization theory, patients with squamous cell carcinoma in the esophagus or head and neck regions are at an increased risk of synchronous and metachronous lesions. Therefore, such patients should undergo endoscopic surveillance for both regions 1 .

However, detailed endoscopic examination of the oral cavity and pharynx is often not performed due to unfamiliarity with the steps and anatomical terms. This could lead to a low rate of early diagnosis of superficial lesions.

In this video, we describe endoscopic pharyngeal evaluation step by step ( Video 1 ).

Endoscopic evaluation of the oral cavity and pharynx: how to do it.Video 1

STEP 1: ORAL CAVITY

Before positioning the mouthpiece, a wide view of the oral cavity is evaluated under white light imaging and narrow-band imaging (NBI). The patient is asked to move the tongue upwards and laterally.

At this point, anatomical landmarks can be observed:

1. hard palate;
2. dorsal surface of the tongue;
3. ventral surface of the tongue;
4. lateral sides of the tongue;
5. buccal mucosa;
6. soft palate.

STEP 2: OROPHARYNX

Still without the mouthpiece, we move on to evaluate the oropharynx. The patient is asked to open the mouth widely and vocalize an “aaaah”.

At this point, anatomical landmarks can be observed:

1. palatopharyngeal arches;
2. uvula;
3. tonsillar pillars;
4. posterior wall of the oropharynx;
5. epiglottis.

STEP 3: HYPOPHARYNX

There is an important blind spot, which is located on the posterior wall of the hypopharynx and postcricoid area. The most important point to adequately observe this region is the Valsalva maneuver using a small mouthpiece that is applied inside the lips 2 . The patient is asked to close the mouth entirely and blow without losing air.

At this point, anatomical landmarks can be recognized:

1. posterior wall of the hypopharynx;
2. piriform sinuses;
3. postcricoid area.

In conclusion, the systematic endoscopic pharyngeal evaluation is feasible ( Fig. 1 ). This should be done routinely in high-risk patients: heavy alcohol drinkers (especially those with “flushing”), heavy smokers, and those with a previous history of esophageal or head and neck cancer who undergo upper endoscopy for other reasons.

**Fig. 1:**
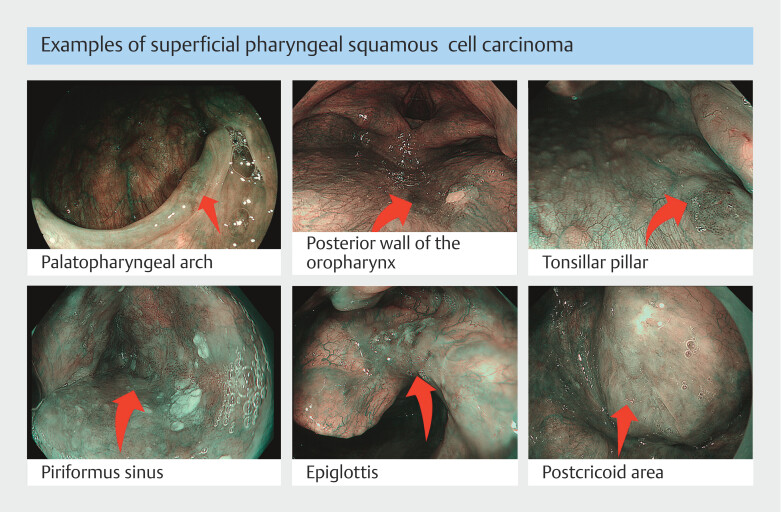
Example of superficial pharyngeal lesions.

Endoscopy_UCTN_Code_CCL_1AB_2AB

## Notes

Endoscopy E-Videos https://eref.thieme.de/e-videos
*E-Videos* is an open access online section of the journal *Endoscopy* , reporting on interesting cases and new techniques in gastroenterological endoscopy. All papers include a high-quality video and are published with a Creative Commons CC-BY license. Endoscopy E-Videos qualify for HINARI discounts and waivers and eligibility is automatically checked during the submission process. We grant 100% waivers to articles whose corresponding authors are based in Group A countries and 50% waivers to those who are based in Group B countries as classified by Research4Life (see: https://www.research4life.org/access/eligibility/ ). This section has its own submission website at https://mc.manuscriptcentral.com/e-videos .

## References

[1] Kato M Ishihara R Hamada K Endoscopic surveillance of head and neck cancer in patients with esophageal squamous cell carcinoma Endosc Int Open 2016 4 E752 755 27556090 10.1055/s-0042-106720 PMC4993894

[2] Iwatsubo T Ishihara R Nakagawa K Pharyngeal observation via transoral endoscopy using a lip cover-type mouthpiece J Gastroenterol Hepatol 2019 34 1384 1389 10.1111/jgh.14574 30561830

